# State-Dependent Decisions Cause Apparent Violations of Rationality in Animal Choice

**DOI:** 10.1371/journal.pbio.0020402

**Published:** 2004-11-23

**Authors:** Cynthia Schuck-Paim, Lorena Pompilio, Alex Kacelnik

**Affiliations:** **1**Zoology DepartmentUniversity of OxfordUnited Kingdom

## Abstract

Normative models of choice in economics and biology usually expect preferences to be consistent across contexts, or “rational” in economic language. Following a large body of literature reporting economically irrational behaviour in humans, breaches of rationality by animals have also been recently described. If proven systematic, these findings would challenge long-standing biological approaches to behavioural theorising, and suggest that cognitive processes similar to those claimed to cause irrationality in humans can also hinder optimality approaches to modelling animal preferences. Critical differences between human and animal experiments have not, however, been sufficiently acknowledged. While humans can be instructed conceptually about the choice problem, animals need to be trained by repeated exposure to all contingencies. This exposure often leads to differences in state between treatments, hence changing choices while preserving rationality. We report experiments with European starlings demonstrating that apparent breaches of rationality can result from state-dependence. We show that adding an inferior alternative to a choice set (a “decoy”) affects choices, an effect previously interpreted as indicating irrationality. However, these effects appear and disappear depending on whether state differences between choice contexts are present or not. These results open the possibility that some expressions of maladaptive behaviour are due to oversights in the migration of ideas between economics and biology, and suggest that key differences between human and nonhuman research must be recognised if ideas are to safely travel between these fields.

## Abstract 2

Decisions made by European starlings in certain choice contexts are shown to depend on the training regimes rather than being inherently irrational behavior

## Introduction

The study of animal behaviour has often incorporated concepts from economic theory. This was the case, for instance, with the introduction of game theory to the study of animal conflict (Maynard-Smith and Price 1973; Maynard-Smith 1974). Similarly, optimal foraging theory (Charnov 1976; Stephens and Krebs 1986) was based on viewing animals as maximisers, with utility often being replaced by rate of energy gain as a proxy for Darwinian fitness, and natural selection playing the role of the short-sighted architect of the decision mechanisms followed by individuals. The foundation for this migration of ideas between fields is the notion that optimal choice is defined by the value of the consequences of each option, and that this value is jointly determined by the option's properties and the chooser's state. This is clear within models, but presents considerable difficulties for empirical tests, and we address some of these problems in this paper.

One consequence of expecting individuals to behave as if they maximised the expected value of a particular function (say, inclusive fitness) is captured in the economic concept of rationality. Since “rationality” is used with very different meanings in different fields (see Kacelnik [2004] for a discussion of rationality and its meanings), it is important to point out that here we will use the term only in its economic sense. Rationality, in this restricted sense, encapsulates several principles that are necessary conditions for the existence of a scale of value consistent across contexts (Mas-Collel et al. 1995). Transitivity, for instance, is a hallmark of rational choice theories. It states that if “a” is preferred to “b”, and “b” to “c”, then “a” should also be preferred over “c”. If, say, “c” were to be preferred to “a”, it would not be possible to place the three options on an ordinal scale. Another principle included in economic rationality is that of independence from irrelevant alternatives (IIA; Arrow 1951), namely, the expectation that preference between a pair of options should be independent of the presence of inferior alternatives. There are different versions of IIA, depending on how demandingly one defines “preference”. A strong probabilistic version, known as the “constant-ratio rule” (Luce 1959), states that the relative proportion of choices made between two options should be the same (as opposed to merely maintaining the same order), regardless of whether they are on their own (binary choices) or in the presence of a third (less preferred) option (trinary choices). A weaker version, known as “regularity”, states that rationality is violated if the proportion of choices for any preexisting option is increased after the addition of a new alternative to the choice set (Luce and Suppes 1965).

Breaches of rationality are well documented in observational or experimental studies on human choice (Tversky 1969; Huber et al. 1982; Payne et al. 1992; Simonson and Tversky 1992; Tversky and Simonson 1993; Wedell and Pettibone 1996; Gigerenzer et al. 1999), and have forced a reinterpretation of much of the existing data and models. In many studies, these violations are taken to imply context-dependent valuation, namely the notion that the (subjective) value of each option is not determined only by its properties and consequences, but instead is constructed at the moment of choice as a function of the number and nature of other options available—a finding used, for example, in marketing and political campaigning for manipulating consumer preferences through the strategic presentation of products and candidates.

An alternative view (Kacelnik and Krebs 1997; Gigerenzer et al. 1999) is that although these mechanisms can cause costly choices, they (the mechanisms) are evolutionarily and/or ecologically rational, meaning that on average in the environment where they evolved or were individually acquired they generate stochastically optimal outcomes. Whichever the interpretation, however, locally costly deviations from rationality do occur, and can offer significant insights in the development of theoretical models of decision-making.

A number of psychological mechanisms have been proposed to explain the effect of inferior alternatives on choice and other examples of irrationality. According to them, the observed failure to exhibit consistent preferences across contexts would be attributable to the dependence of the information-processing mechanisms used by individuals, or of the heuristics used for making choices, on the nature of the choice problem and available alternatives (Shafir et al. 1989; Wedell 1991; Payne et al. 1992). While normative microeconomic theory is independent of process and focuses on revealed preferences, these developments relate the theory to cognition and give weight to the process by which agents reach decisions (Kahneman and Tversky 2000).

It is worth remembering that consistency of preference is accepted by all parties to be only relevant when constancy in the state of the subjects and in the properties of the options is assumed. A subject that prefers lamb to ice cream before dinner, ice cream to coffee immediately after dinner, and coffee to lamb a few minutes later is not considered to be showing intransitivity or violating any principle of rationality, because she is (trivially) changing state between the choices. Similarly, a subject that takes a mango when presented with a basket of many mangoes and only one apple, but takes an apple when faced with equal numbers of both fruits may not be considered irrational, because the value of an option may change when it is the last one, as there is a reputation cost of being impolite and taking the last available fruit of any kind (Sen 1997). Many human experiments are comparisons between groups of subjects that can be assumed to be in equal states at the time of testing but, as we shall see, this is often not ensured in nonhuman animals.

Violations of rationality by animals have also been reported (Shafir 1994; Hurly and Oseen 1999; Waite 2001a; Bateson 2002; Bateson et al. 2002, 2003; Shafir et al. 2002). If these observations are corroborated and found to be systematic, the predictive power of the normative approach to animal behaviour should be questioned. Additionally, the observation of similarly irrational behaviour by animal and human subjects raises the possibility that the same cognitive mechanisms or processes operate in both cases. In fact, explanations for irrationality based on phenomena such as regret and overconfidence (e.g., Loomes and Sugden 1982), proposed with humans in mind, could be tested by examining whether the same circumstances elicit the expression of the same type of paradoxical behaviour in human and nonhuman subjects. If they do, and the mechanisms seem unlikely to operate in nonhuman agents, one may be advised to seek alternative explanations that work well for all kinds of subjects.

Although these possibilities make the study of rationality valuable, critical procedural differences between the two fields have not been sufficiently acknowledged. One crucial distinction derives from the fact that, while human subjects can be verbally instructed about the properties of the alternatives, animals must be exposed to the contingencies to experience or learn about them. This difference hinders the comparison of the mechanisms underlying human and animal choices, since repeated exposure to different contexts often affects the organism's state, thus removing the justification for expecting transitivity, regularity, or any other principle of consistency. In the case of foraging research, different contexts can alter the subjects' net rate of intake during training, so that at the time of choice the resulting state differs and it may be unjustified to expect consistency of preferences. The fact that optimal decisions should be contingent upon state (Houston and McNamara 1999) has been indeed an essential part of normative modelling in biology. As a consequence, apparent violations of rational principles by animals could also result from straightforward state-dependent optimality, the very framework being questioned. It may be added that, although we focus on changes in energetic state that could unwittingly be caused by training, these are not the only possible state consequences of instruction by exposure to the contingencies during training. A subject may be in a different state if the consumption of food items during training affects its nutritional requirements for the achievement of a balanced diet in future choice opportunities (Simpson and Raubenheimer 1993; Raubenheimer and Simpson 1997), or if changes in the context of choice provide it with different information about its future options (Houston 1997).

**Figure 1:**
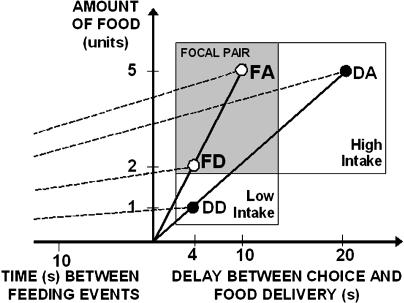
Amount and Delay to Food Corresponding to Each Option The figure shows the parameters of the experiments using the conventional representation used in foraging theory, with energy gains in the ordinate and time in the abscissa. The origin of coordinates is the point of choice, so that time to the right indicates the delay between choice and reward, while time to the left represents all other times in the cycle, in this case the ITI. The options forming the focal choice pair are shown as white circles while those used as decoys are shown by black circles. FA and FD offer the same short-term rate of food intake (slope of the solid lines) of 0.5 units/s, whereas DA and DD offer the same short-term rate of 0.25 units/s. The slopes of the dashed lines (interrupted for space economy) indicate long-term rate of intake, considering the inter-trial interval of 60 s between consecutive feeding opportunities. “High intake” (horizontally adjacent rectangles) and “low intake” (vertically adjacent rectangles) denote the treatments in which decoy DA and DD (or their simulated energetic consequences), respectively, were present in addition to the focal pair. Since DA offers a higher long-term rate of gain than DD, intake is higher in the treatment where DA is present (“High Intake”). The reverse rationale applies to treatment “Low Intake.”

Here we further develop the basis upon which to compare economic rationality between humans and nonhumans, and test whether state-dependent decision-making can be responsible for apparent violations of economic rationality in animal choices. To this end, we compare the foraging preferences of European starlings *(Sturnus vulgaris)* between members of a fixed, focal pair of options across different choice contexts. Our basic paradigm is defined in Figure 1. The members of the focal pair of options differed in that while one of them (focal amount [FA]) offered a higher amount of food, the other (focal delay [FD]) was associated with a better (shorter) delay to food. These two attributes (amount and delay) were counterbalanced between the focal options so as to preserve their ratio (amount/delay), which is known to be (other factors remaining the same) a strong predictor of preference. A third option, or “decoy”, was also available during training and in some of the choice trials. The decoy could be either decoy amount (DA) or decoy delay (DD), depending on treatment (“High Intake” and “Low Intake” respectively, see Figure 1). We refer to the third option as “decoy” because its ratio of amount to delay was lower than in the focal options, and hence it is expected not to be preferred over either (in economic nomenclature, the decoys were “dominated” by the focal options). As postulated by context-dependent (or “comparative”) models of choice (Shafir et al. 1989, 1993; Wedell 1991; Tversky and Simonson 1993), a decoy can potentially affect preferences between a pair of options whenever subjective values are assigned comparatively, namely whenever an option's subjective value depends on the interaction of its properties with those of the remaining alternatives in a set, as well as when the decoy affects a subject's perception of the choice problem. We thus test whether each animal's preference between the focal options changes between two treatments that differed with respect to which of the two decoys was present. To increase comparability with previous research, the parameter values of the decoys were chosen to maximise their putative effect upon preference within the focal pair as postulated by psychological models purported to explain irrational choice (see Materials and Methods for details).

Figure 1 also shows that, although the two focal options and the two decoys were equated in the ratio of amount to delay, they were not equated in terms of their energetic consequences. When all times in the cycle are included, the order in terms of energetic rate of return (the slope of the broken lines in Figure 1) is FA > DA > FD > DD. This means that differences in energetic state as a consequence of training with either of the two decoys could be a confounding factor in interpreting putative differential effects of the decoys. Specifically, repeated exposure to DA could lead to a higher cumulative intake, hence changing choices due to the expression of state-dependent preferences instead of the use of a comparative cognitive mechanism of choice. Our study is aimed at separating these possibilities.

We tested preference between the focal options under three conditions: (1) treatments differing in energetic states when the decoys are absent; (2) treatments differing in which of the two decoys is present, when the energetic consequences caused by each decoy are not controlled; (3) treatments differing in which decoy is present, when the energetic differences they may cause are abolished by supplementary feeding. Our rationale for this design is that if the effects of the decoys are independent of their energetic consequences (i.e., differences in preference between the treatments are observed in all conditions), then these effects may indeed be evidence for a comparative cognitive mechanism of valuation, possibly caused by the same types of cognitive biases and heuristics reported in the human literature. However, if the effects of the decoys are abolished by controlling for energetic consequences and are generated by imposing state changes in the absence of decoys, it would be more parsimonious to explain the effects as state-dependent decision-making. Our results strongly favoured the latter hypothesis.

## Results

### Discrimination of Amounts

**Figure 2:**
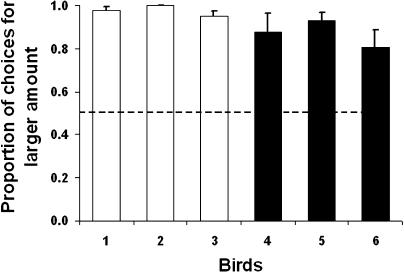
Discrimination Test Proportion of choices (± standard error [s.e.]) made by birds for the option offering the largest amount of food when time parameters were held constant. Choice proportions are significantly different from random for all birds (binomial test, *p* < 0.01). Birds 1, 2, and 3 (white bars) were presented with choices between one and two units of food, and birds 4, 5, and 6 (black bars) with choices between two and five units of food.

We started by testing whether the birds could discriminate between the amounts of reward associated with each of the foraging options shown in Figure 1 (there is already strong evidence that they are able to discriminate between the delays used [Brunner et al. 1992]). Figure 2 shows the proportion of choices of each bird to the option offering the largest amount of food. All birds in both groups significantly preferred the option offering the larger amount (binomial tests, *p* < 0.01 in all cases), confirming that the birds discriminate between these amounts.

### Effects of Intake Rate without Decoys

**Figure 3:**
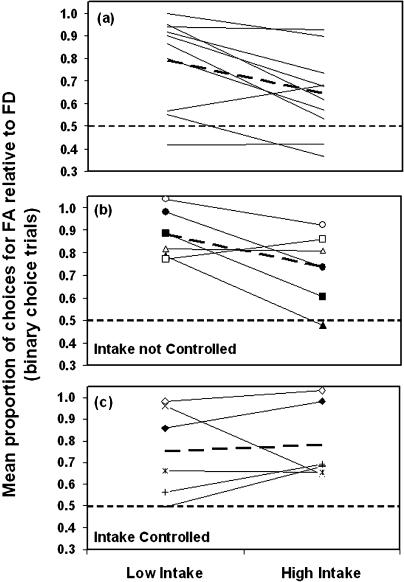
Individual Proportion of Choices for FA Relative to FD in Treatments “High Intake” and “Low Intake” (A) Effect of intake on choices without decoys. Here, extra food simulates the intake consequences that the two decoys cause when they are present and consumed on 25% of the feeding opportunities (*p* < 0.01). (B) Results of an experiment with decoys when energetic consequences of the decoys were allowed to take effect (group NC) (*p* = 0.06). (C) Results of an experiment with decoys, similar to (B), in which the energetic consequences of the decoys were abolished (group C). In (B) and (C), each symbol corresponds to each of the subjects. The dashed lines show the mean values in each of the cases.

The proportion of choices made for each focal option in the absence of decoys when energetic state was manipulated experimentally is shown in Figure 3A. Although the purpose of this experiment was to examine how the strength of preference between the focal options was affected by differences in energetic state, it is worth pointing out that there was an overall preference for FA (the option with higher long-term rate of gain) over FD even though the ratio of amount to delay was the same for both options. This preference for FA is not caused by the accumulated energetic consequences of exposure to each option, because the two focal options were experienced in mixed sessions. In Mazur's (1987) “hyperbolic” model, which is widely used in the behavioural analysis literature, the time between feeding events (or inter-trial interval ) is not included, but instead a constant with the value of 1 s is added to the delay in the denominator. The effect of this term is also to make the value of FA higher than that of FD, consistent with the observed trend.

The critical observation for the present purposes, however, is that the magnitude of the preference between the focal options differed significantly between intake treatments. Specifically, preference for FA over FD was higher in treatment “Low Intake” (the treatment with lower accumulated intake) than in treatment “High Intake” (*F*_1,8_ = 12.1, *p <* 0.008; Figure 3A). The details of the supplementary feeding are given in Materials and Methods, but it is important to highlight that the difference in supplementary intake between treatments “High Intake” and “Low Intake” simulated the differences in state that would be consequent on repeated experience of decoys DA and DD, respectively. These results thus show that energetic state per se can directly affect the strength of preference between alternatives.

The stability criteria (see Materials and Methods) were reached by all but one bird. We therefore also conducted the analysis excluding this bird. The results were the same: Preference for FA over FD was significantly higher in treatment “Low Intake” than in treatment “High Intake” (*F*_1, 7_ = 18.9, *p =* 0.003).

### Test of Economic Rationality in the Presence and Absence of Controls for Intake

In this experiment, two groups of six starlings each (group C had intake controlled between treatments, and NC had intake not controlled between treatments) were trained with three options (the two focal options and one of the decoys) and then allowed to choose between either two (binary trials) or three (trinary trials) of those options. The two treatments, “High Intake” and “Low Intake”, differed in which of the two decoys (DA and DD, respectively) was present during training and in the trinary choices. Within each group, every subject experienced both treatments.

Considering the results of the previous experiment, preference for FA over FD should be higher in the treatment with lower accumulated intake (treatment “Low Intake”) for group NC, in which such intake differences were not eliminated. No differences should, however, be observed in group C, in which intake differences were abolished. The results from the binary choice trials (where only the focal options were present) are shown in Figures 3B and 3C. As predicted, for group NC, preference for FA tended to be higher in treatment “Low Intake” (*F*_1,4_ = 7.4, *p* = 0.06; Figure 3B). In group C, intake differences resulting from the decoys were abolished by supplementary feeding, and no differences in preference between treatments were detected (*F*_1,4_ = 0.2, *p* = 0.677; Figure 3C). To summarise, differences in the level of preference for the focal options in binary choices are present when intake differs but there are no decoys (Figure 3A) and when decoys are present and their intake consequences are not controlled (Figure 3B), but disappear when the decoys are present but their intake consequences are neutralised (Figure 3C).

We also analysed the temporal aspects of state changes on choice. Because the long-term rate of gain offered by DA was higher than that offered by DD, in group NC the difference in cumulative intake between the “High Intake” and “Low Intake” treatments must have increased over the trials in a session. Accordingly, the rate of increase in the strength of preference for FA over FD along the trials (as measured by the slope of the regression of trial number against average proportion of choices for FA) was significantly higher in the latter than in the former treatment (group NC: *F*_1,4_ = 27.35, *p* = 0.006). This difference was not observed for the group in which intake was controlled (group C: *F*_1,4_ = 1.29, *p* = 0.32).

The rational principle of regularity and the constant-ratio rule can be examined by comparing choices between the focal options in binary (only the two focal options present) versus trinary (two focal options and one decoy) trials (see details in Data Analysis). We make two types of comparisons, between-treatments and within-treatments. In the between-treatments comparison we compare the binary trials of one treatment with the trinary trials of the other. For example, we compare the binary trials of treatment “Low Intake” (when training included exposure to FA, FD, and DD, but choices were between FA and FD presented alone) against the trinary trials of treatment “High Intake” (when training included FA, FD, and DA, and choices were between FA, FD, and DA), and vice versa. In the within-treatments comparisons, we compare binary versus trinary trials within the same treatment (e.g., binary versus trinary trials of treatment “Low Intake” and binary versus trinary trials of treatment “High Intake”). Notice that within a given treatment (within-treatment comparisons), accumulated intake was the same in binary and trinary trials for both groups of subjects (C and NC), whereas between treatments (between-treatment comparisons), accumulated intake differed between binary and trinary trials for the group of subjects in which intake differences were not controlled (NC). Therefore, if intake, rather than purely cognitive effects, is the cause of changes in preference for the focal options, apparent violations of regularity and of the constant-ratio rule should be observed only in the between-treatments comparisons for group NC. Conversely, if the presence of the decoys has a cognitive effect upon preferences that is independent of state, such violations should be observed both in the between- and within-treatments comparisons. Table 1 lists the predicted direction of preferences for each of the treatments, considering the hypothesis that differences in intake generated by exposure to the decoys, rather than purely cognitive effects of the decoys, cause the apparent violations of rationality. The directions of preferences were predicted on the basis of the results of the experiment without decoys, which showed that preference for FA was higher in the treatment with lower accumulated intake. Hence, we expect preference for FA to be higher in treatment “Low Intake”; namely, we expect P(FA[“Low Intake”]) > P(FA[“High Intake”]), and consequently, P(FD[“Low Intake”]) < P(FD[“High Intake”]), where P is the strength of preference for the corresponding focal option in the relevant treatment. For simplicity, only the predictions for group NC are shown in Table 1, since under the energetic hypothesis we do not expect differences in preference levels (and therefore violations of rationality) for the group in which intake differences were abolished (group C).

**Table 1:**
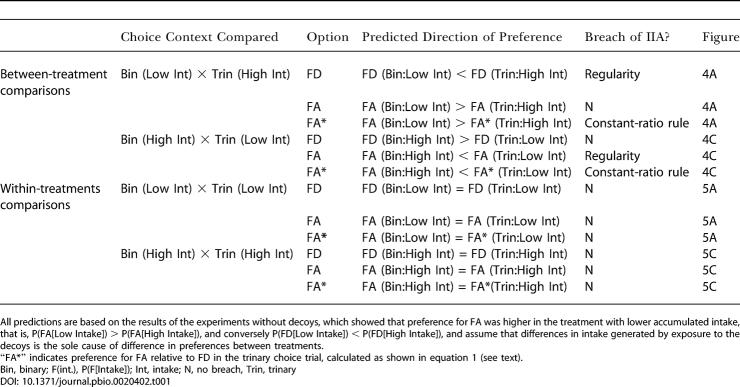
Between- and Within-Treatments Comparison of Binary and Trinary Choice Trials for Group NC

All predictions are based on the results of the experiments without decoys, which showed that preference for FA was higher in the treatment with lower accumulated intake, that is, P(FA[Low Intake]) > P(FA[High Intake]), and conversely P(FD[Low Intake]) < P(FD[High Intake]), and assume that differences in intake generated by exposure to the decoys is the sole cause of difference in preferences between treatments

“FA*” indicates preference for FA relative to FD in the trinary choice trial, calculated as shown in equation 1 (see text)

Bin, binary; F(int.), P(F[Intake]); Int, intake; N, no breach, Trin, trinary

**Figure 4:**
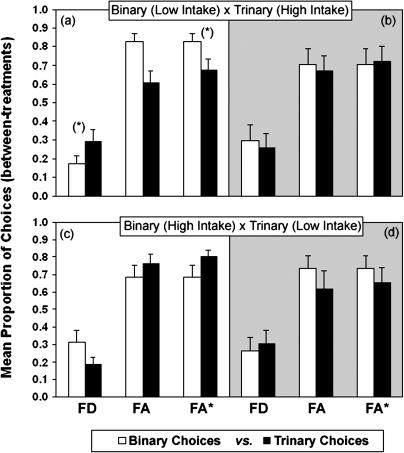
Between-Treatments Comparison in Binary and Trinary Choice Trials The bars show the mean (± s.e.) absolute (FD and FA: leftmost and centre pairs of columns in each panel, respectively) and relative (FA*: rightmost pair of columns in each panel) proportion of choices for each option in binary (white bars) and trinary (black bars) trials when intake rate is not controlled (group NC: A and C; white background) or is controlled (group C: B and D; grey background). Relative preferences were calculated using equation 1 (see text). We compared the preference between the same two focal options between the binary context of one treatment (e.g., treatment “High Intake”) and the trinary of the other (e.g., treatment “Low Intake”). In group C (B and D), none of the differences between binary and trinary contexts were statistically significant. For group NC (A and C), the asterisk (*) indicates a significant violation of either regularity or the constant-ratio rule at *p* < 0.05.

**Figure 5:**
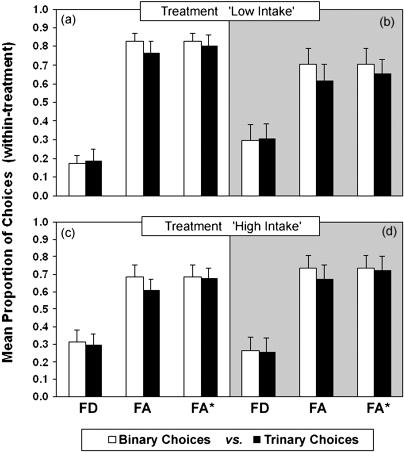
Within-Treatments Comparison of Binary and Trinary Choice Trials The bars show the mean (± s.e.) absolute (FD and FA) and relative (FA*) proportion of choices for each option in the binary (white bars) and trinary (black bars) trials for group NC (A and C) or group C (B and D). Relative preferences were calculated using equation 1 (see text). We compared the preference for the same options between the binary and trinary contexts of the same treatment (when there are no differential energetic effects). There were no violations of either regularity or the constant-ratio rule (*p* > 0.1).

Figures 4 and 5 show the results for the between- and within-treatments comparisons, respectively. The left panels in both figures show the results for group NC (Figures 4A, 4C, 5A, and 5C) and the right panels for group C (Figures 4B, 4D, 5B, and 5D). No violations of either regularity or differences in relative choice proportions were observed in group C (repeated-measures ANOVA, all *p* > 0.1). For group NC, the observed directions of preferences were, in all cases, consistent with all predictions shown in Table 1. In terms of the significance of the observed changes in preference in the between-treatment comparisons (Figure 4A and 4C), one out of the two predicted apparent violations of regularity was statistically significant: There was a significant increase in the absolute proportion of choices for an option (FD) in the trinary with respect to the binary context (F_1,4_ = 7.8, *p* = 0.049; Figure 4A). The constant-ratio rule (see Data Analysis in Materials and Methods) was also violated as predicted in Table 1, because the preference for FA relative to FD was significantly higher in the binary than in the trinary context (F_1,4_ = 9.2, *p* = 0.039; Figure 4A). Finally, against the hypothesis that the effect of decoys on preferences were caused by purely cognitive processes of comparison, there were no significant differences in preferences in the within-treatments comparisons of binary versus trinary trials (repeated-measures ANOVA, all *p* > 0.1; Figure 5A and 5C).

The presence of apparent violations when the effect of the decoys on intake was allowed and their absence when the effect was abolished was again consistent with the hypothesis that these apparent irrationalities were caused by differences in intake brought about by exposure to the decoys. The hypothesis is further confirmed by the absence (both for the group that received supplementary feeding and the one that did not receive it) of violations in the within-treatment comparisons, when the cognitive effect of the decoys was allowed but state was neutralised.

In group NC, all birds reached the stability criteria. In group C, however, one bird did not reach the criteria in treatment “Low Intake”, and another did not reach stability in treatment “High Intake”. We therefore reanalysed the data for this group excluding these two birds. The results were the same, namely, in none of the tests for this group was rationality breached.

## Discussion

Our aim in this study was to foster the development of a solid interdisciplinary basis upon which to compare research on economic rationality in humans and nonhumans, and to investigate whether normatively inspired hypotheses of animal behaviour may be systematically misleading, as they implicitly assume rationality. To this end, we examined whether violations of economic rationality in animals that have recently been reported in the literature represent real violations of rationality caused by the use of comparative cognitive mechanisms of choice as proposed for humans or, alternatively, to unwittingly imposed differences in the state of the subjects. To do this requires testing whether one can reproduce the reported breaches of rationality and whether they are abolished when cognitive effects are allowed but state differences are eliminated. A further test requires generating such violations by changes of state alone. We have achieved all of these conditions in our experiments.

Why should preferences be modulated by energetic state? To start dealing with this question, it is necessary to start by considering why choices do not go exclusively to the option with maximum value. From an evolutionary perspective, one possibility is that subjects are adapted to some level of ambiguity (for instance, because the properties of options may change with time), and tracking these properties requires some level of response to each available option (Houston et al. 1982). If partial preferences are taken as a given, the next stage is to model the factors that may affect them quantitatively. Here, it is possible that partial preferences depend on the benefits that could be derived from each of the available options and that these benefits depend on the state of the subject. To capture this possibility, the probability of choosing a suboptimal action (in this case FD, which offers a poorer long-term rate of gain) could be modelled as a function of the difference between the benefit accruing from each option while the subject is in a given state. For example, inspired by the “matching law” from behavioural analysis (Herrnstein 1961), Kacelnik (1984) tested the fit of a model termed “profitability matching” for starlings experiencing the conditions of the “marginal value” foraging model. In the model, each strategy is deployed in proportion to the ratio of its payoff relative to the sum of the payoffs of all available alternatives. Functionally, such a strategy, while failing to maximise rate of return, may often approximate the optimal strategy or at least avoid costly deviations from it. As highlighted by other authors (McNamara and Houston 1987), more frequent deviations from the optimal policy should be expected when their costs are smaller.

**Figure 6:**
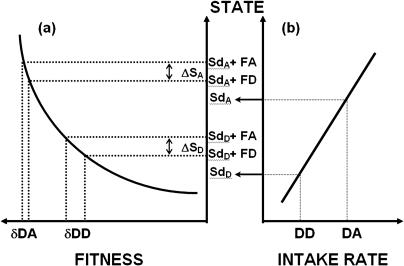
A Functional Model of How State Can Affect Partial Preferences (A) Fitness is plotted as a concave function of the organism's state. Exposure to DD leads to a poorer state (Sd_D_) than that reached after exposure to DA (Sd_A_) (see also B). Sd_D_ + FD and Sd_D_ + FA denote the state reached by subjects under treatment “Low Intake” as a consequence of choosing focal options FD and FA, respectively. Similarly, Sd_A_ + FD and Sd_A_ + FA represent the state reached by subjects in treatment “High Intake” after choosing FD and FA, respectively. (B) State is assumed to be a growing, linear function of energy intake. DD and DA represent the average intake rates experienced by subjects that include the decoys with the same names in their diet. Although choosing FA is always better than choosing FD, and the difference between the states caused by this choice is the same under either treatment (Sd_D_ and Sd_A_), the fitness difference between choosing FA and FD is higher under treatment “Low Intake” (δDD) than “High Intake” (δDA). This should lead to a higher level of preference for FA in the former treatment if choices of the low-yielding option were to be reduced in proportion to their cost.

In Figure 6, we build on this assumption and on the “state” model proposed by Kacelnik and Marsh (2002; see also Marsh et al. 2004) and extend it to illustrate the putative effects of variations in state. We assume that repeated exposure to treatments offering lower and higher objective intake rates (corresponding to decoys DD and DA, respectively) causes some correlated measure of state to be higher in the latter case (Figure 6B). That is, state is assumed to be a positive function of rate of intake during the period preceding the choice itself. We then consider the improvement in state produced by choosing either of the targets (FA and FD). The difference between the improvement in state (ΔS) caused by choosing FA over FD is the same under both treatments, but the biological consequences may differ in magnitude if benefit is not linearly related to state. For the conditions experienced by the starlings in our experiment (where deprivation was very mild), it is reasonable to assume that biological gains were a decreasing function of their initial state (e.g., the contribution of a food item decreases with increasing reserves; see also McNamara and Houston 1982). Figure 6A illustrates this relationship. The figure shows that the cost of choosing the target option with lower long-term rate of energetic gain (FD) is more severe in treatment “Low Intake” than in “High Intake” (|δDD|>|δDA|). Preference for FA should thus be higher under treatment “Low Intake” if the frequency of choices for the leaner focal option is inversely related to their cost. This model is consistent with the equivalence between the effect of supplementary feeding and that of the decoys.

From a mechanistic perspective, it is also possible that, under conditions of higher energetic intake, animals are less motivated to search and work for food. Our data support this possibility. The time subjects took to start working once presented with any option in no-choice trials (i.e., their latency to first peck) was significantly longer in the treatment “High Intake” than in “Low Intake” in the experiment without decoys (*F*_1,8_ = 24.6, *p* = 0.01) and in the experiment with decoys, but only for the group of subjects for which intake was not controlled (group NC: *F*_1,4_ = 39.3, *p* = 0.003; compare this result to that for group C: *F*_1,4_ = 3.7, *p* = 0.13). It is thus possible that these potential differences in motivational state led subjects to pay less attention to the alternatives during a choice opportunity in the treatment “High Intake”, resulting in the observed differences in preference levels.

Within this framework, we now use two examples to consider whether unwittingly induced changes in intake between contexts could also underlie previously reported findings of irrational behaviour.

### Energetic State and Rationality in Jays

A recent study tested the effect of background context on the foraging preferences of semi-tame food-hoarding grey jays (Waite 2001a). The jays were initially split into two groups, and each group was given 25 binary choices in only one of two backgrounds: In background context A, the jays had to choose between one and three raisins placed 0.5 m inside separate tubes (where distance into the tubes should correlate with perceived risk); in B, jays chose between two identical options, each offering one raisin 0.5 m inside the tubes. Both groups were subsequently presented with the choice between one raisin 0.3 m into one tube and three raisins 0.7 m into another tube. In violation of IIA, context had an effect: Preference for the option offering more raisins at a greater perceived risk was higher in the group that had experienced context B, a result interpreted as consistent with the existence of cognitive biases leading to departures from value maximisation (Waite 2001a). However, experience with the two background contexts and consequent differences in amount of food hoarded (i.e., level of energy reserves for future use) between groups could also have led to the observed results. Those jays that had been in context A had collected approximately 62 raisins, whereas those in context B had collected an average of 25 raisins. Assuming that the state of the jays was such that fitness increased following a decelerated function of accumulated raisins, it is possible that those jays previously presented with lower food supplies had more to gain by choosing the option yielding the larger amount of food. Equating their hoards with energy reserves, one could say that they were “hungrier” in context B and hence afforded greater risks to pick the maximum reward. This trade-off between energetic state and predation risk has been extensively discussed within the behavioural ecology literature (e.g., Houston and McNamara 1999)

### Energetic State and Rationality in Hummingbirds

The comparison between binary and trinary choices sometimes employed in studies designed to test economic rationality in animal behaviour can also lead to changes in state. For example, Bateson et al. (2002) compared preferences of Rufous hummingbirds *(Selasphorus rufus)* among three flower types differing in volume and concentration of sucrose (target: 15 μl, 40% sucrose; competitor: 45 μl, 30%; decoy: 10 μl, 35%) in binary (target and competitor) and trinary (target, competitor, and decoy) contexts. The birds experienced both contexts consecutively. In each of them, they made repeated choices between the available flower types until a minimum number of 150 choices for the target and competitor had been reached. The strength of preferences for the competitor over the target increased significantly in the presence of the decoy (trinary context), and the authors interpreted these results as being inconsistent with the use of absolute evaluation mechanisms as normally postulated by functional accounts of behaviour. Yet, the resulting differences could have been caused by exposure to energetically different contexts. Net rate of energy intake of the target, competitor, and decoy were, respectively, 81.9, 92.0, and 59.5 J/s. Considering, for instance, an average proportion of choices for the decoy of about 20% (as described in the study), subjects would necessarily experience a higher intake rate in the binary than in the trinary context unless they modified the relative allocation of responses. Therefore, if the interval between consecutive foraging bouts did not differ systematically between contexts, cumulative gain along the 150 choices would have been lower in the trinary context, favouring preference for the option offering the higher net rate of intake, as reported. Further analyses on the extent to which state differed between contexts, testing for differences in inter-bout intervals, variability in choices, and unplanned differences in nutrient balance (i.e., in the actual volumes and concentration of sugar and water; see, for example, Simpson and Raubenheimer 1993) experienced in each context in this and other experiments with hummingbirds (e.g., Bateson et al. 2003) are therefore needed before concluding that the results imply violations of rationality rather than compensations for differences in state generated by the introduction of a decoy.

These examples were provided to illustrate that acknowledging and controlling for the effects that differences between choice sets may produce on an organism's state is paramount when investigating the influence of context on choice behaviour. This is particularly important because it is often difficult to predict how changes in state will affect preferences. For instance, our results showed a higher level of preference for the larger but more delayed reward when the starlings were under a poorer schedule—a result also previously reported by some authors (Christensen-Szalanski et al. 1980; Rechten et al. 1983). Conversely, results showing an effect of state in the opposite direction have also been reported in the literature and interpreted as demonstrating greater impulsivity under hungrier conditions (Snyderman 1983; Lucas et al. 1993). From a functional viewpoint, whether lowering energetic state should shift preference towards bigger and later rewards over smaller and more immediate ones or vice versa depends on the details of the problem. While many authors dealing with the problem of temporal discounting focus on one-shot choices, animal experiments are conducted in repeated trials, where delays mean lost opportunity, and where consideration of variance (“risk”) must come into the picture. For example, when the pressing factor is maximisation of rate of intake, greater ITIs have two effects: They alter the state of the subjects (lowering their energy reserves), and they shift the difference in long-term rates in favour of larger, more delayed rewards (a result that may mistakenly be considered a decrease in impulsivity). On the other hand, when risk is the main factor, it is impossible to make a general prediction, because the consequences of variance in both size and delay to reward are functionally sensitive to the curvature of the fitness versus state function, and this is likely to have one or more inflexion points.

Because the directionality of state effects under biologically rational choice is difficult to predict, to demonstrate the presence of true breaches of rationality or to confirm previous findings as evidence of irrationality using these experimental economics paradigms, it is therefore essential not only to investigate the immediate effects of state on preference, but also to ensure that these violations are reproduced and not altered in any direction when state is controlled. Additionally, the observation that rewards received under higher states of need often lead to faster acquisition (Capaldi and Havancik 1973; Tarpy and Mayer 1978; Balleine 1992) makes it fundamental to control for differences in state whenever subjects have to learn the properties of the rewards.

### Conclusion

Following the growing body of claims for irrational choice behaviour by human subjects, recent reports on breaches of rationality in animals may be interpreted as questioning the predictive power of the optimality approach in behavioural ecology, favouring the view that the reported inconsistencies result from rigid rules of evaluation and choice leading to the assignment of context-dependent values to options and devaluing the contribution of functional reasoning. We do not doubt that the precise empirical description of decision rules is important. Indeed, findings of locally irrational behaviour are useful tools for the investigation of the mechanisms underlying choices, often forcing a reinterpretation of existing data and models of optimal decision-making. Additionally, the potential dependence of valuation mechanisms on the context of choice might have direct implications for other biological systems. For example, Shafir et al. (2003) have recently emphasised the role of pollinator perception and choice strategies in mediating the evolution of floral nectar distribution strategies, as well as the potential use of knowledge of cognition-mediated mechanisms of choice on the development of biological control programs. Still, if ideas are going to travel safely between economics and biology, crucial details of the experimental paradigms must be scrutinised, and differences between human and nonhuman research must be acknowledged. Here we emphasise that, due to the need of exposing animals to the contingencies of the choice problem, contextual changes may lead to variations in the state of individuals, which in turn can affect both the amount of knowledge acquired by subjects and the parameters of the decision faced by the individuals, thus calling into question the significance of apparent violations of rational axioms.

We do not claim that state dependence accounts for all reported inconsistencies in animal choice (e.g., Waite 2001b; Shafir et al. 2002), nor are we suggesting that animal choices are based directly upon calculations of optimal state-dependent actions instead of direct psychological mechanisms of choice. Indeed, the notion of “rules of thumb” that perform well in most relevant ecological situations, but may also lead to suboptimal behaviour, has been long accepted in behavioural and evolutionary biology, and may well also comprise some of the comparative mechanisms of choice and “fast and frugal” heuristics previously described for human beings (e.g., Gigerenzer et al. 1999). However, we believe that if evolutionarily inspired normative models of behaviour are to be treated fairly, a deep scrutiny of the causes underlying observations of apparent economic irrationality in animal (and, for that matter, human) choices should be attempted. Economic theory has been and still is a source of inspiration for optimality theorising in biology, and experimental economics may just as well inspire understanding of the predictive failure of some of these models. Conversely, the systematic observation of local cases of irrationality in animals may provide insights into the nature of the mechanisms of choice employed by humans. However, our study highlights that at least some apparent similarities in the expression of “maladaptive” behaviours may be due to oversights in the implementation of experiments testing ideas that originate in other disciplines.

## Materials and Methods

### 

Our main experiment consisted of training starlings to choose between either three or two simultaneously presented foraging options. Each option was implemented as a coloured, intermittently flashing key that, when pecked once by the subject, caused the other keys to darken, stopped flashing, and then delivered a certain amount of food following the first peck after a programmed delay. The amount and delay to food determined the features of each option. In total, there were four options in the experiment, two forming a “focal pair” of target options and two that were called “decoys”.

#### Parameters of the options

The actual reward parameters corresponding to the four options are shown in Figure 1. The two focal options, FA and FD, offered a ratio of amount of reward to delay to reward of 0.5 food units per second, while each of the two decoys, DA and DD, offered a ratio of 0.25 units per second (the slopes of the solid lines in Figure 1). We call these ratios “short-term rates” for consistency with previous literature (viz., Bateson and Kacelnik 1996). Short-term rate is known to be strongly correlated to attractiveness, but it is not a description of objective intake rate, because it does not include times other than the delay between choice and outcome. This difference is important and underlies this study. Functional approaches to foraging behaviour, such as classical optimal foraging theory (Stephens and Krebs 1986), have highlighted that energetic gains are a function of total intake over total time. This relationship is expressed by defining the value of an option by its real rate of returns as given by A/(D + ITI), where A is food amount, D is the delay between choice and outcome, and ITI, is the sum of all other times in the foraging cycle. This expression, known as “long-term rate”, is the slope of the broken lines in Figure 1. Any consideration of the effect of energy state on preferences must consider long-term rate, even if the subjects used only short-term rates to form preferences. Scholars concerned with the mechanisms by which stimuli acquire significance (and hence potential attractiveness) to a learning animal, such as the behavioural analysis and conditioning literatures, have focused on the conditions that make the association between the outcome and the predictive event easier. In this case, the predictive event is the onset of the stimulus marking the delay to food, which coincides with the animal's action of pecking at it (Green et al. 1981; Kacelnik 1984; Mazur 1987; Bateson and Kacelnik 1996).

We programmed the alternatives so that the two focal options were as close as possible to being equally attractive and superior to the two decoys, themselves equated in short-term rate. At the same time, we used the fact that the energetic consequences of the two decoys are very different to manipulate energetic consequences.

The parameters of the decoys were chosen to maximise their putative cognitive effect on preferences between the focal options. Several accounts of the effect of poorer alternatives have proposed that they may have an effect because decision-makers compare each attribute of the options (in our case amount and delay) independently, not integrated into a single expression of value. In the conditions described by Figure 1, two putative mechanisms could cause DA to increase preference for FA. One of them, referred to as the comparative model (Shafir et al. 1989; Wedell 1991; Shafir et al. 1993; Tversky and Simonson 1993), postulates that DA could favour FA by means of its asymmetric relationship of dominance (with dominance used as a synonym for superiority) with the focal options. The overall idea is that an option gains value when it is better than other options in the set along a particular attribute. In the present case, DA is dominated by FA in one attribute (delay) and equal in the other (amount), but it is dominated by FD in one attribute (delay) while it dominates it in the other (amount). Thus, if subjects are influenced by the number of relationships of dominance between attributes, FA could be more attractive than FD for being the only option that dominates DA completely. A second mechanism of interest, known as the “range effect”, says that the same difference in a physical attribute can have a greater effect when embedded in a narrower range of values (Parducci 1965). Therefore, the quantitative advantage of an option along a given attribute decreases as a function of the range of values present. For example, if only FA and FD are present, the range of delays is 6 s, and FD's advantage is 100%. When, say, DA is added, the range increases to 16 s, and FD's advantage over FA is only 37.5% of the total range. Since the range in amounts is not modified by adding DA, this again may favour FA. The same two mechanisms could make DD enhance the attractiveness of FD over FA. Note, however, that although these mechanisms provide a possible direction for which differences in preferences could be observed, changes in preference levels between contexts are usually interpreted as being compatible with context dependence regardless of their direction and the number of attributes describing the options (Hurly and Oseen 1999; Bateson 2002; Bateson et al. 2002).

#### Subjects.

Subjects were 28 naïve starlings captured in Oxford (English Nature licence # 20020068). After capture, the birds were kept outdoors and, during the experiment, transferred to individual indoor cages (120 cm × 60 cm × 50 cm) that served as housing and testing chambers. Lights were on between 0500 and 1900 h, and temperature ranged from 12 °C to 16 °C. Subjects were visually but not acoustically isolated. The experiments took place between June and October 2002.

#### Apparatus

Each cage had a panel with a central food hopper and three response keys. Computers running Arachnid language (Paul Fray, Cambridge, UK) served for control and data collection. Rewards were units of Orlux pellets, crushed and sieved to an even size (0.025 ± 0.005 g). Automatic pellet dispensers (Campden Instruments, Leicester, UK) delivered rewards at a rate of 1 unit/s. Each option was signalled by a different key colour (red, green, yellow, blue, white, or pink).

#### Experimental protocol

Subjects were first trained to peck at keys to obtain rewards until all birds pecked at least 80% of the food opportunities. A discrete trials procedure with two types of trials (no-choice and choice) was then employed. No-choice trials provided the birds information about each alternative, but also contributed to their rate of intake. These trials started with one key blinking. The first peck caused the light to stay steadily on. The first peck after the programmed delay had elapsed triggered the delivery of the programmed amount of food, followed by a fixed ITI of 60 s, during which all keys were off. Since food was delivered following the first peck after the end of the designated delay, experienced delays were slightly longer than those programmed (median [interquartile range] in experiment with decoys: treatment “Low Intake”, delay FD = 4.1 s [0.2 s], delay FA = 10.2 s [2.0 s], delay DD = 4.1 s [0.2 s]; treatment “High Intake”, delay FD = 4.1 s [0.2 s], delay FA = 10.2 s [1.8 s], decoy DD = 20.2 s [0.3 s]).

Choice trials began with two or three keys (depending on whether choice was binary or trinary) simultaneously blinking. The first peck on any of them caused the pecked key to turn steadily on and the others to turn off. After that, the trial continued as in no-choice trials. The order and sides in which the options were presented was randomised. After the sessions, subjects were fed ad libitum with turkey crumbs for at least 2 h, then supplemented with ten mealworms, and then food deprived until the beginning of the experimental sessions on the following morning.

In all experiments (except the calibration for discrimination of amounts) we used a within-subjects design with two treatments. The within-subjects design was preferred owing to the high level of variability between individual starlings' energetic requirements, which would have prevented accurate control of energetic state between groups. We therefore focus our analyses on within-subjects comparisons, since variations in the energetic state of subjects between groups of different subjects would hinder the comparison of their preference levels. The pairing of options with colours was balanced across subjects and changed between treatments. Treatment order was balanced across birds. Subjects were given one resting day with ad libitum food between treatments. Each treatment lasted for 20 sessions. Data from the last five sessions were used for analyses.

#### Discrimination of amounts

In the discrimination experiment, we used a between-subjects design with six male starlings, split into two groups of three birds each. Group 1 chose between 1 and 2 units of food, and group 2 chose between 2 and 5 units. Rewards were delivered after the first peck on the corresponding key. Subjects experienced two sessions per day, at 0800 and 1300 h. Each session consisted of 84 trials, divided into 21 blocks of four trials each: two no-choice (each option presented once) followed by two choice trials.

#### Effects of intake rate on choice without decoys

To investigate the potential effect of different intake rates on preference between the target options, we used ten birds (five males and five females) in a within-subjects design with two treatments, which differed with respect to the amount of supplementary food delivered to the starlings. One of the treatments simulated the intake effect (total amount of food consumed) of experience with decoy DA (treatment “High Intake”) on 25% of the foraging opportunities (this proportion was established from the average proportion of trials in which the decoy was experienced in a pilot study). The other treatment simulated the intake effect of experience with decoy DD (treatment “Low Intake”) on 25% of all foraging opportunities. To achieve this we delivered unconditionally the reward corresponding to the appropriate decoy once per experimental block (details below), after the ITI that followed the last trial of that block. Unlike the trials with the pair of focal options, no action was needed on the part of the subjects to receive the unconditional reward, nor was any specific discriminative stimulus associated with it.

There were three daily sessions, at 0600, 1000, and 1400 h. Each session consisted of 36 trials, grouped into 12 blocks of three trials each. Each block started with two no-choice trials (each focal option once), followed by an ITI and an unconditional food delivery in which the amount of the simulated decoy was delivered after the delay corresponding to that decoy had elapsed. The third and last trial of each block was a choice between the two focal options.

#### Test of economic rationality in the presence and absence of controls for intake

Twelve birds were randomly assigned to two groups (intake controlled, or group C, and intake not controlled, or group NC) of six birds each (three males and three females in each group). All subjects experienced two treatments, one with decoy DA (treatment “High Intake”) and another with DD (treatment “Low Intake”). In group C, the differences in intake rate between treatments caused by the exposure to the energetically different decoys were eliminated with supplementary feeding.

Three daily sessions started at 0500, 0900, and 1400 h. Each session consisted of 63 trials, grouped into seven blocks of nine trials each: three no-choice trials followed by six choice trials in a random order (two trinary choices, two binary choices between the focal options, and two binary choices between each focal and the decoy). To equalise intake between treatments in group C, we adopted the following procedure. We calculated the maximum obtainable amount of food and delay per block for treatment “High Intake” (the treatment offering the higher cumulative delay and amount), and in both treatments delivered supplementary rewards up to this amount and delay twice per block. Thus, in every block of trials we equalised intake and total time to the same value in both treatments. The supplement was delivered after the fourth and the ninth trial of each block, after adding the appropriate delay to the ITI. In both treatments, supplements in the middle of the block were followed by a 5-min no-food interval to prevent satiation. Blocks were separated by 10-min intervals.

#### Data analysis

According to the principle of IIA, the strength of preference between two options should be independent of the presence of other (less preferred) options. These other options may either form part of the general situational background and be absent at the time of the choice, or form part of an enriched set of options at choice time. To test whether differences in background led to breaches of IIA, we compared choice proportions in binary choices (in which the two target options of the focal pair were paired) between the two treatments by conducting separate tests for each group of subjects. To test the temporal effects of potential state changes over the trials in the experimental sessions (i.e., whether the strength of preference between the focal options changed along a session), we calculated the slope of the regression of trial number against (transformed; see below) proportion of choices for FA over FD and tested whether the group of slopes was different between treatments for both groups of subjects.

We also tested for differences in preference between the focal options across contexts, comparing binary with trinary choice trials. We performed two analyses. First, we tested whether the relative strength of preference between the focal options differed between the binary and trinary contexts. Relative preferences were calculated as



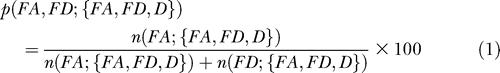



where *p*(FA,FD;{FA,FD,D}) is the relative preference for FA over FD when the alternatives indicated inside the curly brackets were present, and *n*(FA;{FA,FD,D}) is the number of choices for FA within the same set of alternatives. D stands for either of the decoys (DA or DD). The second term in the denominator follows the same notation. According to a strong probabilistic version of IIA known as the constant-ratio rule (Luce 1959), relative preferences should be the same between binary and trinary contexts.

Second, we compared absolute strength of preference for each of the targets between the two contexts to test for violations of regularity. Regularity is a weaker form of IIA (Luce and Suppes 1965), which asserts that the absolute proportion of choices for an option cannot increase when a new option is added to the choice set. Again, breaches of regularity are usually taken as strong evidence that the value of an option is assigned in a context-dependent way (see Schuck-Paim and Kacelnik [2002] for an intuitive explanation).

We used repeated-measures ANOVA on square-root arcsine–transformed choice proportions, having treatment and order as within- and between-subjects factors, respectively. In all cases we tested the effect of order and interaction between the factors, but neither was significant. The assumptions of normality and homogeneity of variances were not violated for any of the transformed datasets. The Greenhouse-Geiser correction was applied whenever the assumption of sphericity was violated. Tests were always two-tailed. In all conditions, we additionally tested whether the birds' preferences were already stable when the experimental sessions were interrupted. We considered preferences to be stable when the regression of choice proportions (for the focal choice pair in binary choices, where only FA and FD were available) in five consecutive sessions (against session number) was not significant and the standard deviation of these proportions did not exceed 0.20.

## Abbreviations

DA: decoy amount
DD: decoy delay
FA: focal amount
FD: focal delay
IIA: independence from irrelevant alternatives
ITI: inter-trial interval
s.e.: standard error
